# Auswirkungen der ersten COVID‑19-Welle auf die Viszeralchirurgie: Ein retrospektiver Fallzahlenvergleich an einem Universitätsklinikum und einem Krankenhaus der Grund- und Regelversorgung

**DOI:** 10.1007/s00104-021-01434-5

**Published:** 2021-05-19

**Authors:** Marcella Steffani, Constanze Merz, Christian Stöß, Lars Landau, Norbert Hüser, Daniel Hartmann, Helmut Friess, Jörg Theisen, Alexander Novotny

**Affiliations:** 1 Fakultät für Medizin, Klinikum rechts der Isar, Klinik und Poliklinik für Chirurgie, Technische Universität München, Ismaninger Straße 22, 81675 München, Deutschland; 2 Abteilung für Allgemein‑, Viszeral‑, Thorax- und Endokrine Chirurgie, Klinikum Landkreis Erding, Erding, Deutschland

**Keywords:** Appendektomie, Cholezystektomie, Chirurgie, Postoperative Komplikationen, SARS-CoV‑2, Appendectomy, Cholecystectomy, Surgery, Postoperative complications, SARS-CoV‑2

## Abstract

**Hintergrund:**

Während der ersten COVID-19-Pandemiewelle führte die Aussetzung aller elektiven Eingriffe im Zeitraum vom 15.03. bis 15.05.2020 in Deutschland zu einem Rückgang an Operationen. Die Auswirkungen auf die Zahl spezifischer Operationen in der Viszeralchirurgie sind bislang nicht bekannt.

**Methoden:**

In diese retrospektive Studie wurden 301 Patienten eingeschlossen, die eine Cholezystektomie bzw. Appendektomie an einem Universitätsklinikum oder Krankenhaus der Grund- und Regelversorgung zwischen dem 15.03.2020 und 05.05.2020 (vs. 2018 und 2019) erhielten. Analysiert wurden die Fallzahlen und der klinische Verlauf.

**Ergebnisse:**

Die Aussetzung des Elektivprogramms führte zu einer signifikanten Reduktion elektiver Cholezystektomien und notfallmäßiger Appendektomien. Am Universitätsklinikum reduzierte sich die Anzahl der Appendektomien von 24 im Jahr 2018 um 33 % auf 16 im Jahr 2020, die Zahl der Cholezystektomien sank von 30 um 57 % auf 13. Am Grundversorger reduzierte sich die Zahl der Appendektomiepatienten von 23 im Jahr 2018 um 48 % auf 12 im Jahr 2020, die Zahl der Cholezystektomien stieg im Jahr 2018 auf 2019 an und sank anschließend um 30 % im Jahr 2020. Die Operationsdauer, Krankenhausverweildauer und der klinische Verlauf der Patienten unterschied sich nicht signifikant zu den Vorjahreszeiträumen.

**Diskussion:**

Der Lockdown während der ersten COVID-19-Pandemiewelle führte zu einer deutlichen Reduktion häufiger viszeralchirurgischer Eingriffe. Um die medizinische Versorgung der gesamten Bevölkerung während einer Pandemie möglichst auf hohem Niveau zu erhalten, müssen aktuelle Möglichkeiten der operativen und konservativen Therapie, unter anderem abhängig von lokalen Infektionszahlen und den individuellen Komorbiditäten der Patienten, gegeneinander abgewogen werden.

## Abstract (en)

**Background:**

During the first wave of the COVID-19 pandemic, the downregulation of all elective interventions during the period from 15 March 2020 to 15 May 2020 in Germany led to a decrease in operations. The impact on the number of specific operations in visceral surgery is so far unknown.

**Methods:**

This retrospective study included 301 patients who underwent a cholecystectomy or appendectomy at a university hospital or a primary care facility between 15 March 2020 and 15 May 2020 (vs. 2018 and 2019). The number of cases and the clinical outcomes were analyzed.

**Results:**

The suspension of elective surgery resulted in a significant reduction in elective cholecystectomy and emergency appendectomy. At the University Hospital the number of appendectomies decreased by 33% from 24 in 2018 to 16 in 2020 and the number of cholecystectomies declined by 57% from 30 in 2018 to 13 in 2020. At the primary care hospital, the number of appendectomy patients decreased by 48% from 23 in 2018 to 12 in 2020 and the number of cholecystectomies increased from the year 2018 to 2019 and subsequently declined by 30% in 2020. The duration of surgery, length of hospital stay and clinical course of patients did not significantly differ from the previous year time periods.

**Conclusions:**

During the first wave of the COVID-19 pandemic, the lockdown resulted in a significant reduction in frequently carried out visceral surgical interventions. In order to maintain the optimal medical care for the total population, current options for surgical and conservative treatment must be weighed against each other depending on the numbers of local infections and the individual comorbidities of the patients.

Seit Dezember 2019 breitet sich das Virus SARS-CoV‑2 („severe acute respiratory syndrome coronavirus type 2“) weltweit aus. Die Pandemie hat dabei große Auswirkungen auf die Krankenhäuser weltweit. In Deutschland sind davon besonders die chirurgischen Kliniken betroffen. Um genügend Kapazitäten für die an COVID-19 („coronavirus disease 2019“) erkranken Patienten zu schaffen, mussten elektive Operationen ausgesetzt werden. In dieser retrospektiven Studie werden die Auswirkungen der ersten COVID-19-Welle auf die Viszeralchirurgie anhand der Fallzahlen und der perioperativen Parameter eines Universitätsklinikums und eines Krankenhauses der Grund- und Regelversorgung dargestellt.

## Hintergrund

Die virusinduzierte Pneumonie „coronavirus disease 2019“ (COVID-19) breitet sich nach wie vor weltweit aus und beeinflusst sowohl das öffentliche Leben als auch den Klinikalltag der Krankenhäuser. Im Dezember 2019 wurde erstmalig in Wuhan das Virus SARS-CoV‑2 als Ursache für die neu aufgetretene Lungenerkrankung identifiziert [1]. Seither steigen die Infektionszahlen kontinuierlich an, sodass am 11.03.2020 die World Health Organization (WHO) den Ausbruch des Virus zur Pandemie erklärte [2].

Um die Neuinfektionen und Letalität zu senken, wurden zahlreiche präventive Maßnahmen zur Begrenzung der Virusausbreitung erlassen [3]. Deutschlandweit wurden durch einen Regierungsbeschluss ab dem 12.03.2020 alle planbaren Aufnahmen, Operationen und Eingriffe auf zunächst unbestimmte Zeit verschoben, um den steigenden Bedarf an Intensiv- und Beatmungskapazitäten zur Behandlung von COVID-19-Patienten decken zu können [4].

Das Ziel dieser retrospektiven Studie ist es, die Auswirkungen der ersten COVID-19-Welle auf die Fallzahlen der Viszeralchirurgie zu untersuchen. Anhand der Appendektomie und Cholezystektomie, die zu den am häufigsten durchgeführten Operationen in der Viszeralchirurgie zählen, wurden die Fallzahlen eines Universitätsklinikums (Klinikum rechts der Isar, München) sowie Krankenhauses der Grund- und Regelversorgung (Klinikum Erding, Landkreis Erding) verglichen [5]. Im Zeitraum des Lockdowns stiegen in der Stadt München die Infektionszahlen von 256 COVID-19-positiven Patienten am 15.03. auf 6240 infizierte Menschen am 15.05. (Prävalenz 423/100.000 Einwohner; [6]). Insgesamt wurden 203 COVID-19-assoziierte Todesfälle gezählt. Im Landkreis Erding stieg die Infektionszahl von 35 auf 547 COVID-19-positiv getestete Bürger (400/100.000 Einwohner). 11 Personen verstarben an COVID-19 [6].

## Methoden

### Studiendesign

Eingeschlossen wurden alle Patienten, die eine Appendektomie oder Cholezystektomie während des ersten Lockdowns der COVID-19-Pandemie (vom 15.03. bis zum 15.05.2020) am Universitätsklinikum (Klinikum rechts der Isar, München) oder Krankenhaus der Grund- und Regelversorgung (Klinikum Erding, Landkreis Erding) erhielten. Diese wurden im Vergleich zu den Daten aus demselben Zeitraum (jeweils vom 15.03. bis 15.05.) der Jahre 2018 und 2019 analysiert. Verglichen wurden die Operationsart, die Operationsdauer, der intraoperative und der histopathologische Befund, die Krankenhausverweildauer, Beginn der präoperativen Symptomatik, die notfallmäßige oder elektive Vorstellung und Operationen der Patienten, Drainagenanlage, freie Flüssigkeit präoperativ, postoperative Komplikationen (Clavien-Dindo ≥3a), Entzündungsparameter und die antibiotische Therapie, welche zusammengefasst wurde als intraoperative Einzeldosis und postoperative Therapie.

### Statistische Analyse

Kontinuierliche Variablen werden als Mittelwerte mit Standardabweichung angegeben. Kategoriale Variablen werden als absolute und relative Häufigkeiten beschrieben und mittels χ^2^- oder Fisher’s Exact-Test analysiert. Für metrisch skalierte Variablen wurde der Mann-Withney-U-Test angewendet. Alle statistischen Tests wurden mit SPSS 26.0 (SPSS Inc., Chicago, USA) durchgeführt. Alle *p*-Werte <0,05 wurden als statistisch signifikant gewertet.

## Ergebnisse

### Allgemeine Charakteristika

**Figure 1:**
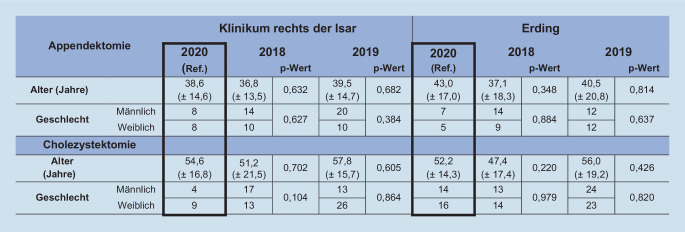

Insgesamt haben in den Jahren 2018 bis 2020 im Zeitraum vom 15.03. bis zum 15.05. am Universitätsklinikum 70 Patienten eine Appendektomie erhalten, 82 Patienten eine Cholezystektomie. Am Krankenhaus der Grund- und Regelversorgung wurde im gleichen Zeitraum bei 59 Patienten eine Appendektomie und bei 107 Patienten eine Cholezystektomie durchgeführt (Abb. 1). Patienten, die eine Appendektomie am Universitätsklinikum erhielten, hatten ein durchschnittliches Alter zwischen 36,8 und 39,5 Jahren; am Grund- und Regelversorger zwischen 37,1 und 43 Jahren. Das durchschnittliche Alter der Patienten, die eine Cholezystektomie erhielten, lag am Universitätsklinikum zwischen durchschnittlich 51,2 und 57,8 Jahren und am Klinikum der Grund- und Regelversorgung zwischen 47,4 und 56 Jahren (Abb. 1).

**Figure 2:**
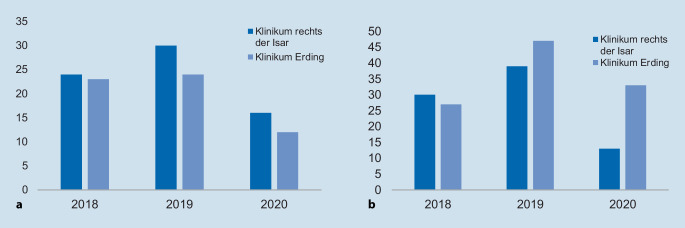

Während der Pandemie nahmen am Universitätsklinikum die Fallzahlen sowohl der Appendektomien als auch der Cholezystektomien ab. 2018 erhielten 24 Patienten eine Appendektomie, 2019 30 Patienten, hingegen wurde 2020 nur bei 16 Patienten eine Appendektomie durchgeführt. Bei der Durchführung der Cholezystektomien wurde während der ersten Pandemiewelle eine Reduktion um 57 % beobachtet (Abb. 2). Auch am Grund- und Regelversorger zeigte sich eine deutliche Abnahme an durchgeführten Appendektomien: 2018 erhielten 23, 2019 24 und 2020 lediglich 12 Patienten eine Appendektomie. Bei den Fallzahlen der Cholezystektomie zeigt sich hingegen ein Unterschied zum Universitätsklinikum: Es zeigte sich ein Anstieg der Operationszahlen von 27 Patienten 2018 auf 47 Patienten 2019, die eine Cholezystektomie erhielten, anschließend eine Reduktion 2020 auf 33 Patienten (Abb. 2).

### Appendektomie – Analyse der perioperativen Parameter

**Figure 3:**
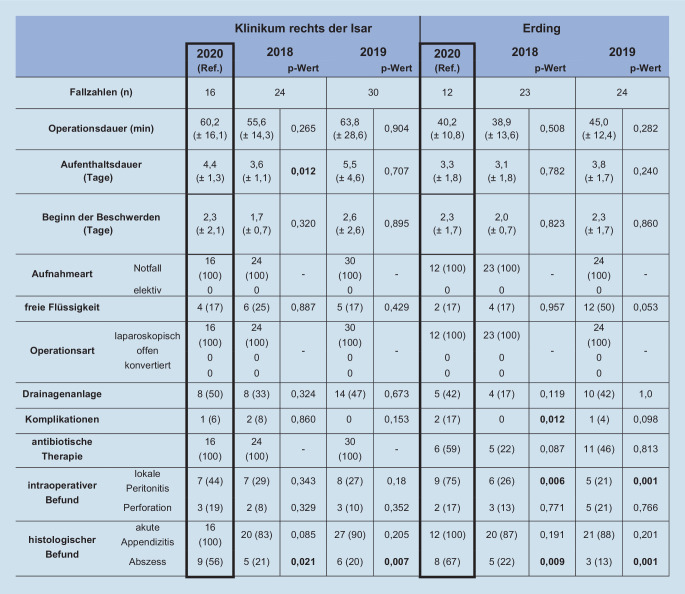

Alle Appendektomien wurden an beiden Kliniken notfallmäßig durchgeführt. Appendektomien am Universitätsklinikum dauerten im Jahr 2020 im Vergleich zu 2018 mit durchschnittlich 60,2 (±16,1) zu 55,6 (±14,3) Minuten länger. Am Grund- und Regelversorger stieg die Operationsdauer von 38,9 (±13,6) im Jahr 2018 auf 40,2 (±10,8) Minuten im Jahr 2020 an. Drainagen wurden an beiden Kliniken vermehrt angelegt: Am Universitätsklinikum stieg der Anteil um 17 % (2018: 33 %; 2020: 50 %), am Grundversorger wurden 25 % mehr Drainagen angelegt (2018: 17 %; 2020: 42 %). An beiden Kliniken stellten sich im Untersuchungszeitraum 2020 alle Patienten notfallmäßig vor. In keinem Fall musste konvertiert werden. Am Universitätsklinikum erhielten alle Patienten eine antibiotische Therapie. Am Klinikum der Grund- und Regelversorgung stieg die Gabe von Antibiotika von 22 % (5/23) 2018 auf 59 % (6/12) 2020 an. Die stationäre Aufenthaltsdauer am Universitätsklinikum stieg von 3,6 (±1,1) Tagen im Jahr 2018 auf 4,4 (±1,3) Tage im Pandemiejahr an. Präoperativ begannen die Beschwerden der Patienten im Jahre 2020 im Mittel bereits 2,3 (±2,1) Tage vor der Appendektomie, 2018 1,7 (±0,7) Tage zuvor. Bei beiden Kliniken wies der histopathologische Befund auf eine geringere Rate an negativen Appendektomien hin. An beiden Kliniken stieg der Anteil an komplizierten Appendizitiden zudem im Jahr 2020 signifikant an: Am Universitätsklinikum zeigte sich ein Anstieg um 35 % (*p* = 0,007), am Grund- und Regelversorger ein Anstieg um 54 % (*p* = 0,001). Für das Klinikum der Grund- und Regelversorgung zeigte sich kein Unterschied zwischen den Jahren hinsichtlich der stationären Aufenthaltsdauer sowie der Dauer der präoperativen Beschwerden. Postoperativ zeigte sich kein Anstieg der Komplikationen am Universitätsklinikum. Für den Grund- und Regelversorger ergab sich ein leichter Anstieg (2019: *n* = 1, 2020: *n* = 2; Abb. 3).

### Cholezystektomie – Analyse der perioperativen Parameter

Bei den Cholezystektomien stieg am Universitätsklinikum die durchschnittliche Operationsdauer von 75,7 (±26) im Jahr 2018 und 80,4 (±30,8) im Jahr 2019 auf 84,2 (±21,4) Minuten im Jahr 2020. Auch die Krankenhausverweildauer stieg von 4,7 (±3,0) auf 5,6 (±3,7) Tage im Jahr 2020. Am Grund- und Regelversorger zeigte sich kein relevanter Unterschied in der Operations- oder stationären Aufenthaltsdauer.

Bezüglich der präoperativen Beschwerden lässt sich bei beiden Kliniken ein deutlicher Anstieg der akuten Beschwerden erkennen: Am Universitätsklinikum gaben 2018 30 % (9/30) der Patienten an, seit 1 bis 7 Tagen und 57 % (17/30) der Patienten seit über 28 Tagen an Beschwerden zu leiden (*p* = 0,02). 2019 begannen bei 15 % (6/39) der Patienten die Beschwerden 1 bis 7 Tage und bei 67 % (26/39) der Patienten über 28 Tage präoperativ (*p* = 0,001). Hingegen litten 2020 54 % (7/13) der Patienten seit 1 bis 7 Tagen und nur 8 % (1/13) seit über 28 Tagen vor der Operation an Beschwerden. Somit wurden während des ersten COVID-19-Lockdowns deutlich mehr akute Cholezystitiden operiert. Am Klinikum der Grund- und Regelversorgung stellten sich 2018 37 % (10/27) der Patienten mit weniger als einer Woche bestehenden Beschwerden vor und 52 % (14/27) gaben an, seit über 28 Tagen Beschwerden zu haben. Im Unterschied dazu litten 2020 insgesamt 61 % (20/33) der Patienten seit 1 bis 7 Tagen an Beschwerden und 27 % (9/33) seit über 28 Tagen.

An beiden Kliniken wurden die Cholezystektomien überwiegend laparoskopisch durchgeführt. Am Universitätsklinikum wurden 2018 80 % (25/30) laparoskopisch und 11 % (2/30) offen operiert. Konvertiert wurden 9 % (3/30) der Cholezystektomien. 2019 wurden 77 % (30/39) der Patienten laparoskopisch, 21 % (8/39) offen operiert und 3 % (1/39) konvertiert. Im Jahr 2020 stieg die Anzahl der laparoskopischen Cholezystektomien auf 92 % (12/13), 8 % (1/13) wurden offen durchgeführt. Am Grund- und Regelversorger erfolgte sowohl 2018 als auch 2020 die Cholezystektomie bei allen Patienten laparoskopisch, 2019 wurde bei einem Patienten konvertiert (2 %).

Histopathologisch wurden am Universitätsklinikum signifikant mehr akute Cholezystitiden diagnostiziert (*p* = 0,005). Hiermit einher gehen signifikant weniger chronische Cholezystitiden (*p* = 0,006). Im Grund- und Regelversorger zeigten sich keine signifikanten Unterschiede zwischen den Vergleichsjahren.

Auch bei der Aufnahme- und damit Operationsart zeigt sich eine deutliche Veränderung der Vergleichsjahre zu 2020: Am Universitätsklinikum stellten sich 2018 20 % (7/30) der cholezystektomierten Patienten notfallmäßig vor, 80 % (28/30) der Patienten wurden elektiv operiert (*p* = 0,05). Ähnlich stellte sich dies 2019 dar. 2020 wurden 62 % (7/13) notfallmäßig operiert. Am Grund- und Regelversorger zeigte sich auch ein Anstieg der notfallmäßigen Vorstellungen, es wurden jedoch weiterhin elektive Cholezystektomien durchgeführt: 2018 und 2019 stellten sich jeweils ca. 50 % der Patienten notfallmäßig und 50 % elektiv vor. 2020 stieg die notfallmäßige Vorstellung der Patienten auf 79 % (26/33) an.

**Figure 4:**
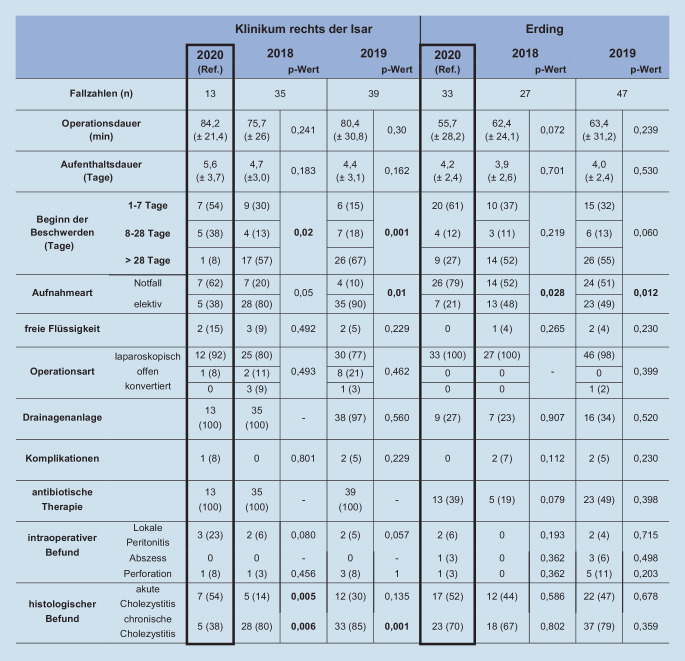

Präoperativ wurden am Universitätsklinikum zunehmend akute Cholezystitiden sonographisch nachgewiesen (2018: 9 %, 2020: 15 %). Am Klinikum der Grund- und Regelversorgung zeigte sich kein Unterschied bezüglich dieses Kriteriums. In beiden Kliniken ergaben sich sowohl bei der Häufigkeit intraoperativ eingelegter Drainagen als auch der Anzahl an Komplikationen und antibiotischer Therapie keine signifikanten Unterschiede zwischen den Vergleichsjahren (Abb. 4). Im Jahr 2020 wurde ein Patient am Grund- und Regelversorger nach dringlicher Cholezystektomie positiv auf eine COVID-19-Infektion getestet. Weitere COVID-19-positive Patienten gab es in beiden Kohorten nicht.

## Diskussion

In der vorliegenden Studie konnte gezeigt werden, dass die Fallzahlen sowohl am Universitätsklinikum als auch am Grund- und Regelversorger für Notfalleingriffe wie der Appendektomie deutlich zurückgegangen sind (Abb. 2). Am Universitätsklinikum konnte zudem eine Reduktion von elektiven bzw. frühelektiven Operationen am Beispiel der Cholezystektomie beobachtet werden (Abb. 2). Wie erwartet reduzierte sich die Zahl elektiv geplanter Cholezystektomien, gleichzeitig erhöhte sich die Zahl notfallmäßiger Indikationsstellungen, welche mit einer erhöhten Anzahl an akuten Beschwerden einherging. Am Klinikum der Grund- und Regelversorgung konnte hingegen keine signifikante Reduktion der Cholezystektomien beobachtet werden. Es zeigten sich sowohl bei der Appendektomie als auch bei der Cholezystektomie keine erhöhten Komplikationsraten, jedoch ließ sich am Universitätsklinikum eine steigende Tendenz der Operationszeit, Drainagenanlage und stationärer Aufenthaltsdauer bei appendektomierten Patienten beobachten. Dies geht mit einer erhöhten Anzahl an akuten komplizierten Appendizitiden einher.

Wie in den Ergebnissen gezeigt und in der breiten Öffentlichkeit wahrgenommen, wurden in der Phase des ersten Lockdowns in Deutschland deutlich weniger Patienten operiert. Die Studie zeigt anhand konkreter Fallzahlen, wie sich die bundesweite Aussetzung des Elektivprogramms auf die Viszeralchirurgie auswirkt, um Krankenhauskapazitäten zu schaffen [4]. Auch in anderen Ländern zeigte sich ein ähnlicher Rückgang an Operationen [7]. Zudem zeigte eine Umfrage unter den Ordinarien für Viszeralchirurgie in Deutschland, dass eine Reduktion der Operationen an den Uniklinika wahrgenommen wurde [8]. Dies bestätige sich auch in der Analyse des Berufsverbands Deutscher Chirurgen über alle chirurgischen Fachdisziplinen und Bundesländer hinweg: Der Rückgang an Operationen betrug im März und April 2020 zwischen 18 und 49 % im Vergleich zum Vorjahreszeitraum [9].

Die Reduktion der elektiven Operationskapazitäten zeigt sich deutlich am Beispiel der Cholezystektomien. Am Universitätsklinikum Klinikum rechts der Isar reduzierten sich die Cholezystektomien um 57 % von 2018 zu 2020. Zudem wurde keine der Cholezystektomien elektiv durchgeführt. Alle Cholezystektomien wurden als Notfalleingriffe durchgeführt. Auch am Klinikum Landkreis Erding zeigt sich eine Reduktion der Cholezystektomien um 30 % von 2019 auf 2020.

Im Vergleich zum Klinikum Erding zeigte sich die Operationsdauer am Klinikum rechts der Isar zur Zeit der COVID-19-Pandemie deutlich verlängert. Dies könnte damit zusammenhängen, dass vermehrt akute Cholezystitiden mit fortgeschrittenen Befunden operiert wurden (histologisch stieg die Rate an akuten Cholezystitiden signifikant an). Zudem können akute Cholezystitiden mit ungünstigeren Operationsbedingungen assoziiert sein. Hierzu passt, dass die Krankenhausverweildauer der am Universitätsklinikum operierten Patienten anstieg.

Der Grund für die deutlich niedrigere Anzahl an notfallmäßigen Appendektomien bei Patienten mit akuten Beschwerden lässt sich schwieriger erfassen. Wie Tschaikowsky et al. im Rahmen ihrer deskriptiven epidemiologischen Studie zeigten, haben sich die Fallzahlen in den Notaufnahmen während des Lockdowns signifikant reduziert. Es ist insbesondere ein Rückgang an Patienten mit abdominellen Beschwerden (23 %) im Vergleich zum Vorjahreszeitraum zu beobachten [10]. Auch Nourazari et al. berichten in ihrer retrospektiven Studie von einem Rückgang der notfallmäßigen Fallzahlen in den USA [11]. Die Verunsicherung der Patienten und Meidung der Notaufnahmen ist vermutlich, zusätzlich zu den politischen Maßnahmen, mitverantwortlich für den Rückgang an Operationen bzw. Appendektomien. Die gesunkene Zahl an Appendektomien während der Pandemie ist vergleichbar mit anderen internationalen Studien. So zeigten Tankel et al., dass die Zahl der Appendektomien in Israel zum Zeitpunkt des Lockdowns deutlich reduziert war. Der perioperative Verlauf war ebenso wie in der vorliegenden Untersuchung im Vergleich zu den Vorjahren unverändert [12]. Als Erklärung für diese Ergebnisse werden von den Autoren unter anderem die Bewegungseinschränkungen der Bevölkerung und die Angst vor Infektionen im Krankenhaus genannt.

Um während der Corona-Pandemie die medizinische Versorgung auf hohem Niveau zu sichern, muss der Einsatz operativer und konservativer Behandlungen gegeneinander abgewogen werden. Collard et al. haben hierzu beispielsweise einen Algorithmus entwickelt, um akute unkomplizierte Appendizitiden bei Erwachsenen primär antibiotisch zu behandeln. In die Entscheidungsfindung hinsichtlich der Primärtherapie (Appendektomie vs. Antibiotika) müssen sowohl diagnostische Befunde (z. B. eine Computertomographie) als auch die Komorbiditäten des Patienten mit einfließen [13]. Bei einem initial konservativen Vorgehen müssen die Patienten jedoch immer darüber aufgeklärt werden, dass die Rezidivrate nach 5 Jahren bei 40 % liegt [14]. In der CODA-Studie (Comparison of the Outcomes of antibiotic Drugs and Appendectomy) zeigte sich eine Operationsrate nach primärer konservativer Therapie mit Antibiotikagabe von 29 % [15]. Eine alternative Strategie für den ambulanten Sektor beschreiben Lefrançois et al. [16]. Anhand der Saint-Antoine Scale (Body-Mass-Index <28 kg/m^2^, Leukozyten <15.000/μl, C‑reaktives Protein <30 mg/l, Bildgebung ohne Hinweis auf eine akute komplizierte Appendizitis) wird die Chance für einen unkomplizierten Verlauf eingeschätzt, um zwischen primär antibiotisch oder chirurgischer Therapie zu entscheiden.

### Limitationen

Die vorliegende retrospektive Studie weist mehrere Limitationen auf. Hinsichtlich der Komplikationen erhielten Patienten aus der Kohorte des COVID-19-Pandemiejahres ein kürzeres postoperatives Beobachtungsintervall. Dieses kann zu einer falsch-niedrigen Komplikationsrate führen. Zudem wurde insgesamt ein kleines Patientenkollektiv eingeschlossen. Es wird deshalb angestrebt, nach Ende der zweiten COVID-19-Welle einen Vergleich der beiden Lockdownzeiträume durchzuführen.

## Fazit

- Die COVID-19(„coronavirus disease 2019“)-Pandemie hat während des Lockdowns zu einer deutlichen Reduktion elektiver und notfallmäßiger viszeralchirurgischer Eingriffe geführt.
- Es wurden mehr komplizierte Appendizitiden und mehr akute Cholezystitiden operiert.
- Es zeigte sich jedoch kein Anstieg der postoperativen Komplikationen.
- Um die medizinische Versorgung der gesamten Bevölkerung während der Pandemie bei steigenden Infektionszahlen und kritischen Kapazitäten der Krankenhäuser zu erhalten, müssen Möglichkeiten der operativen und konservativen Therapie, unter anderem abhängig von lokalen Infektionszahlen und den individuellen Komorbiditäten der Patienten, gegeneinander abgewogen werden.
